# Supplementary material for: Modular interneuron circuits control motion sensitivity in the mouse retina
Source: Nat Commun. 2023 Nov 27;14:7746. doi: 10.1038/s41467-023-43382-0 (PMC10679153; doi:10.1038/s41467-023-43382-0)
Supplement: Supplementary file 1 — Supplementary Information [file 41467_2023_43382_MOESM1_ESM.pdf]

Table S1. Morphology of COMS-AC and CK2-AC2

	Soma diameter (μm)	Dendritic diameter (μm)	Dendritic stratification (% in IPL)
COMS-AC	7.5 ± 0.1	32.0 ± 0.7 (proximal)<br>46.5 ± 0.9 (distal)	0.10 ± 0.04 (proximal)<br>0.43 ± 0.09 (distal)
CK2-AC2	7.5 ± 0.2	69.0 ± 2.5 (proximal)<br>66.9 ± 2.3 (middle)<br>53.3 ± 2.9 (distal)	0.42 ± 0.08 (proximal)<br>0.65 ± 0.05 (middle)<br>0.83 ± 0.05 (distal)

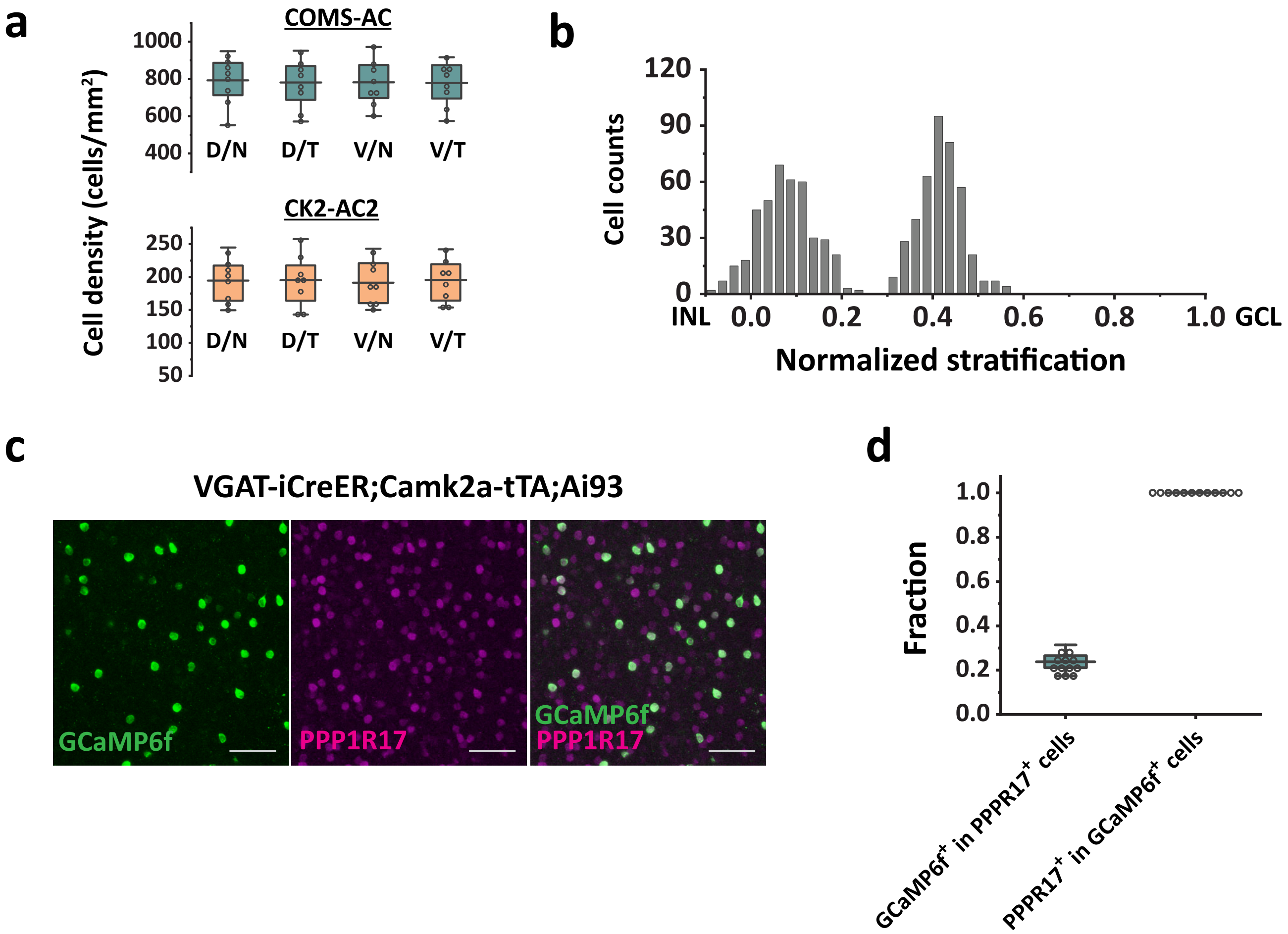

Supplementary Figure 1. Single cell type labeling in VGAT-iCreER;Camk2a-tTA;Ai93 with tamoxifen injected after P100. a. Densities of GCaMP6f-labeled cells in the four quadrants of VGAT-Cre;Camk2a-tTA;Ai93 mice, n = 8 retinae. D/N: dorsal/nasal, D/T: dorsal/temporal, V/N: ventral/nasal, V/T: ventral/ temporal. **b.** Stratification of GCaMP6f-labeled cells from 10 retinas is in line with the exclusive labeling of bi-stratified COMS cells (n = 412 cells, peaks: 7.5% ± 0.2%, 41.7% ± 0.1%, mean ± SEM). Cells were randomly selected from each retina. **c.** All GCaMP6f-labeled cells were positive to immunostaining of PPP1R17. Scale bar, 50μm. **d.** A portion of PPP1R17-expressing cells (23.8% ± 1.2%) are labeled with GCaMP6f, whereas all GCaMP6f-labeled cells exhibit positive PPP1R17 expression, n = 12 retinae. **b, c,** and **d,** Experiments were performed in VGAT-iCreER;Camk2a-tTA;Ai93 mice with tamoxifen injected after P100. The box plots display the mean, 25th, and 75th percentiles, while the whiskers indicate the 1.5 interquartile range. Source data of **a, b,** and **d** are provided as a Source Data file.

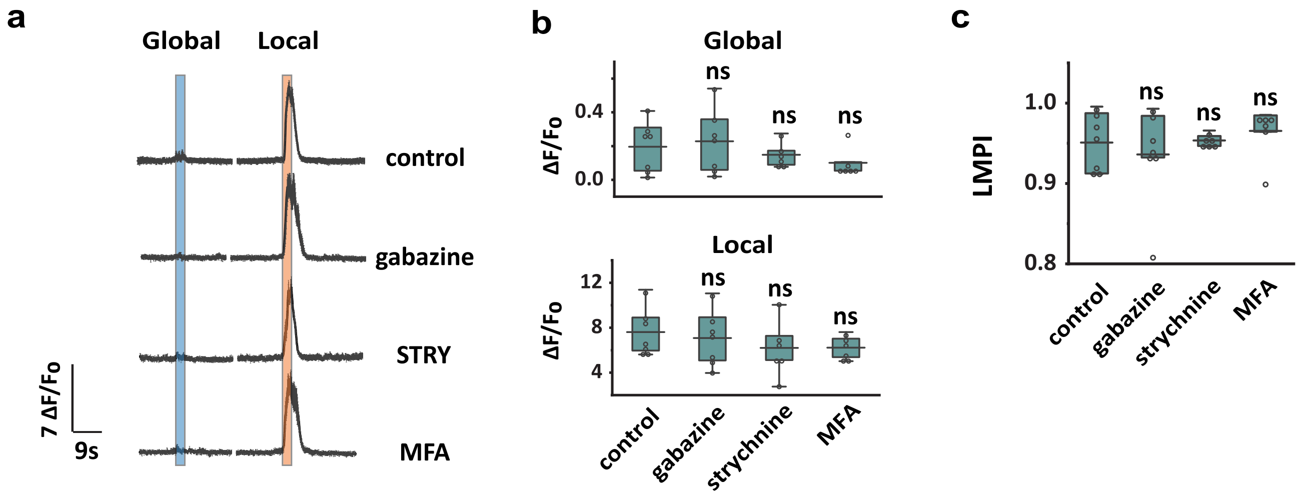

Supplementary Figure 2. Gabazine, strychnine, and MFA have no effects on the object motion sensitivity of COMS-AC. a. GCaMP6f responses to global and local motion in gabazine, strychnine, or MFA. **b.** Summarized effects of gabazine, strychnine, and MFA on GCaMP6f responses to global motion (top) and local motion (bottom). N = 7 cells for control and gabazine, n = 6 cells for strychnine and MFA. Global: ns: $p = 0.58$ for gabazine, $p = 0.44$ for strychnine, $p = 0.16$ for MFA. Local: ns: $p = 0.22$ for gabazine, $p = 0.22$ for strychnine, $p = 0.16$ for MFA, Wilcoxon Signed Rank test, two tailed. **c.** Summarized effects of gabazine, strychnine, and MFA on LMPI. LMPI was calculated from **b.** N = 7 cells for control and gabazine, n = 6 cells for strychnine and MFA. ns: $p = 0.69$ for gabazine, $p = 0.56$ for strychnine, $p = 0.44$ for MFA, Wilcoxon Signed Rank test, two tailed. The box plots display the mean, 25th, and 75th percentiles, while the whiskers indicate the 1.5 interquartile range. Source data of **b** and **c** are provided as a Source Data file.

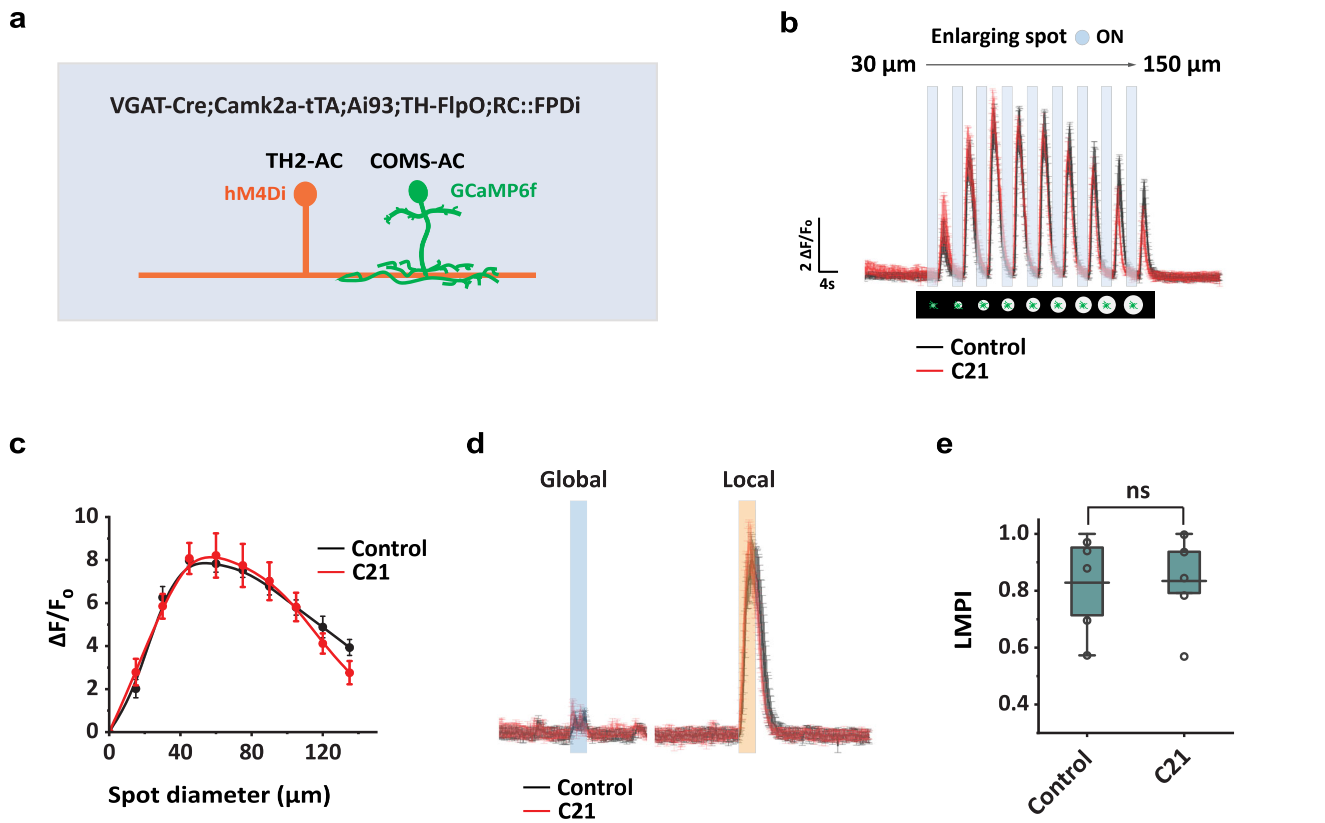

Supplementary Figure 3. Silencing TH2-AC has no effect on the receptive field and OMS of COMS-AC. a. Schematic illustration for expressing hM4Di in TH2-AC while expressing GCaMP6f in COMS-AC with VGAT-Cre;Camk2a-tTA;Ai93;TH-FlpO;RC::FPDi mice. **b.** Probing the receptive field of COMS-AC in control and C21, n = 5 cells. **c.** Receptive field of COMS-AC in control and C21, n = 5 cells. **d.** GCaMP6f responses in global and local motion for control and C21, n = 5 cells. **e.** LMPI of COMS-AC in control and C21, n=5 cells. ns (p = 1), Wilcoxon Signed Rank test, two tailed. The box plot displays the mean, 25th, and 75th percentiles, while the whiskers indicate the 1.5 interquartile range. Source data of **c** and **e** are provided as a Source Data file.

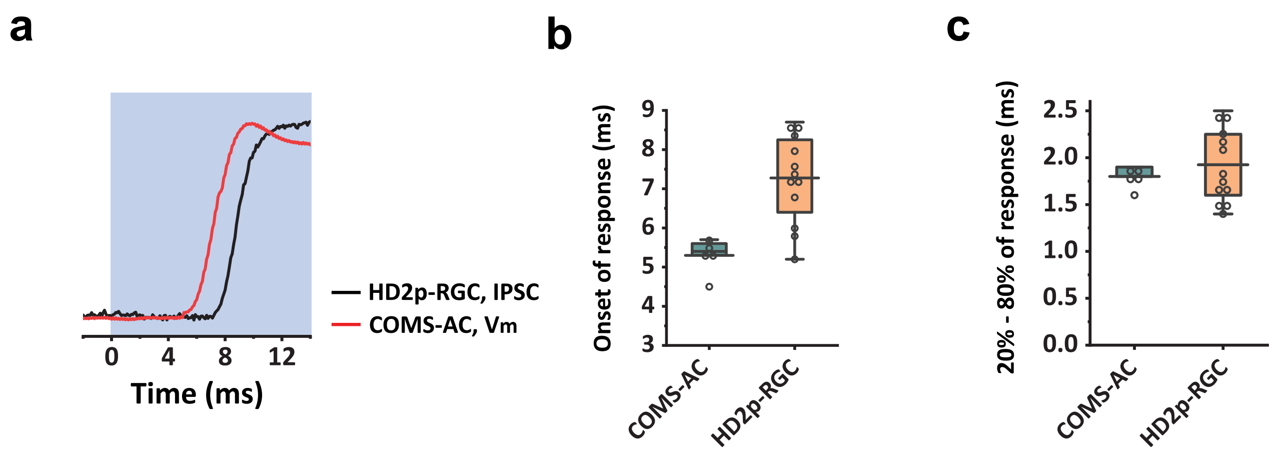

Supplementary Figure 4. Kinetic analysis of ChR2-mediated depolarization in COMS-AC and IPSC in HD2p-RGC. a. Induction of depolarization in COMS-AC and IPSC in HD2p-RGC with ChR2 activation (blue bar). **b.** Time after ChR2 activation stimulus to onset of COMS-AC and HD2p-RGC responses. COMS-AC: 5.3 ± 0.2 ms (n = 5 cells), HD2p-RGC: 7.3 ± 0.3 ms (n = 12 cells). Therefore, the synaptic latency from COMS-AC depolarization to IPSC induction in HD2p-RGC is ~2ms. **c.** Rise time from 20% to 80% of the peak amplitudes for COMS-AC depolarization and HD2p-RGC IPSC. COMS-AC: 1.8 ± 0.1 ms (n = 5 cells), HD2p-RGC: 1.9 ± 0.1 ms (n = 12 cells). The box plots display the mean, 25th, and 75th percentiles, while the whiskers indicate the 1.5 interquartile range. Source data of **a, b,** and **c** are provided as a Source Data file.

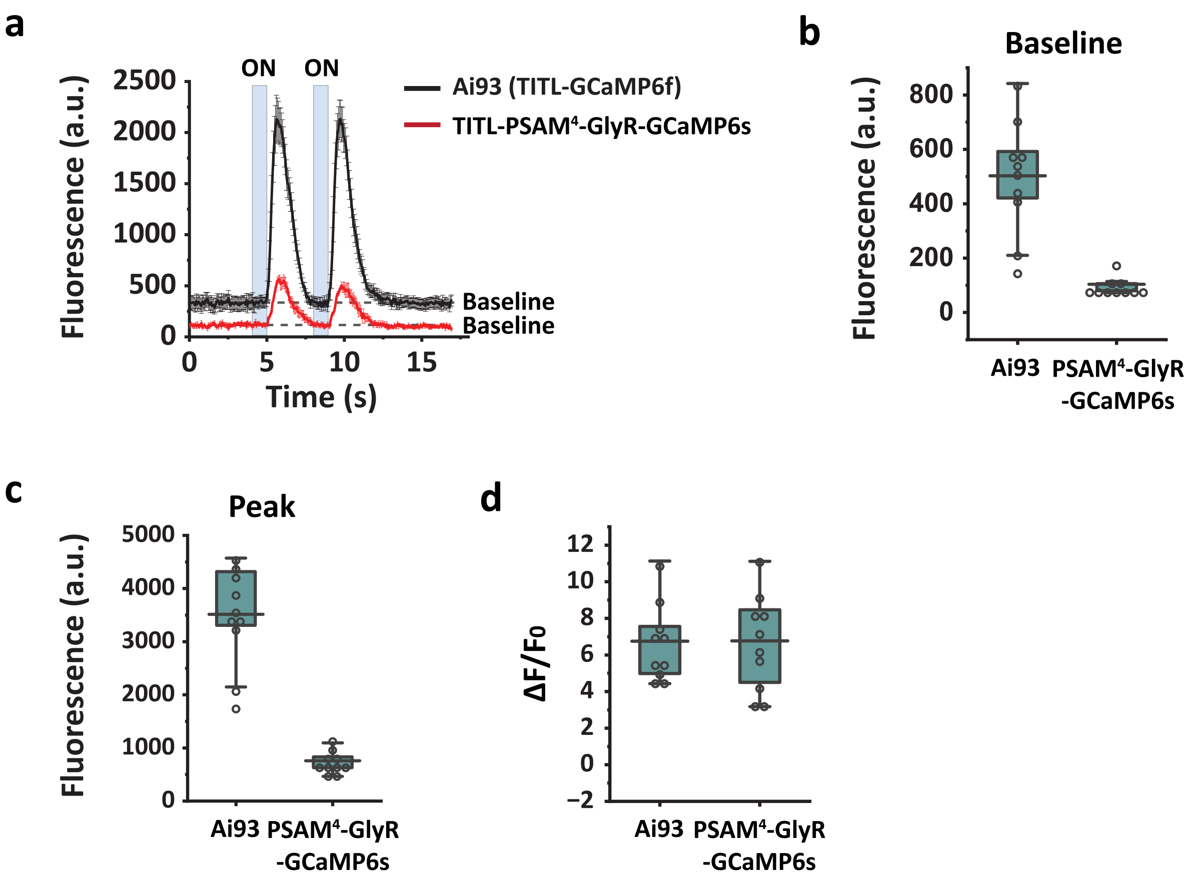

Supplementary Figure 5. Comparison of GCaMP6 signals produced by TITL-PSAM^4-GlyR-GCaMP6s and Ai93 (TITL-GCaMP6f) in COMS-AC. a. GCaMP6 signals in response to spot stimulation (50μm), recorded in VGAT-Cre;Camk2a-tTA;Ai93 mice (gray) or VGAT-Cre;Camk2a-tTA;TITL-PSAM^4-GlyR-IRES-GCaMP6s mice (red), n = 10 cells for both. **b-d.** Comparison of Ai93 and TITL-PSAM^4-GlyR-IRES-GCaMP6s in baseline **(b)**, peak response **(c)**, and $\Delta F/F_0$ **(d),** n=10 cells for both. Compared to Ai93, TITL-PSAM^4-GlyR-IRES-GCaMP6s produced 20.6 % of baseline fluorescence, 21.6% of peak fluorescence, and 100.3% of $\Delta F/F_0$. The box plots display the mean, 25th, and 75th percentiles, while the whiskers indicate the 1.5 interquartile range. Source data of **b, c,** and **d** are provided as a Source Data file.

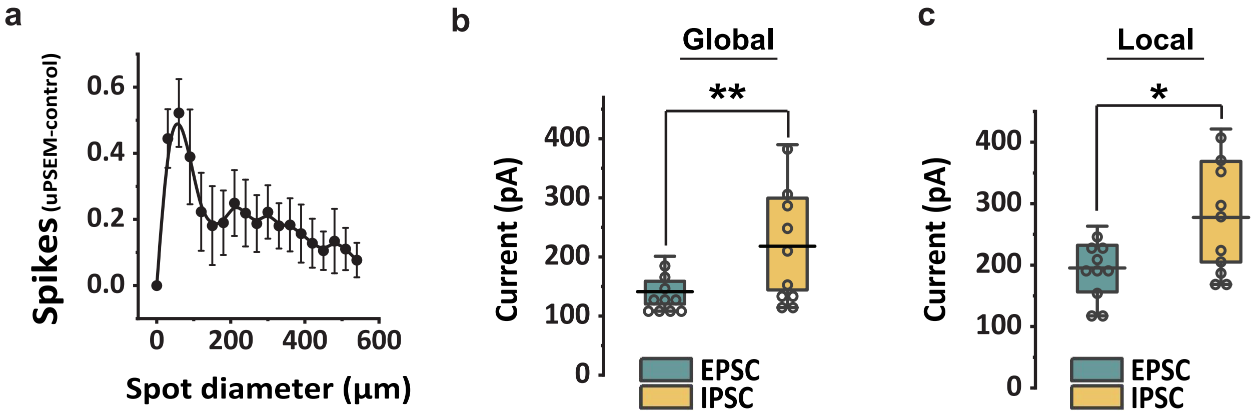

Supplementary Figure 6. Effects of COMS-AC silencing on HD2p-RGC. a. Effects of uPSEM[792] on HD2p-RGC OFF spiking rate in response to spot stimulation. Spikes $_{(uPSEM\text{-}control)}$ = Spikes $_{(uPSEM^{792})}$ - Spikes $_{(control)}$, calculated from Figure 7c. N = 6 cells, error bars represent SEM. **b.** EPSC and IPSC in control HD2p-RGCs during global motion. N = 10, **p = 0.003, Wilcoxon Signed Rank test, one tailed. **c.** EPSC and IPSC in control HD2p-RGCs during local motion. N =10 cells, *p = 0.032, Wilcoxon Signed Rank test, one tailed. The box plots display the mean, 25th, and 75th percentiles, while the whiskers indicate the 1.5 inter-quartile range. Source data of **a, b,** and **c** are provided as a Source Data file.

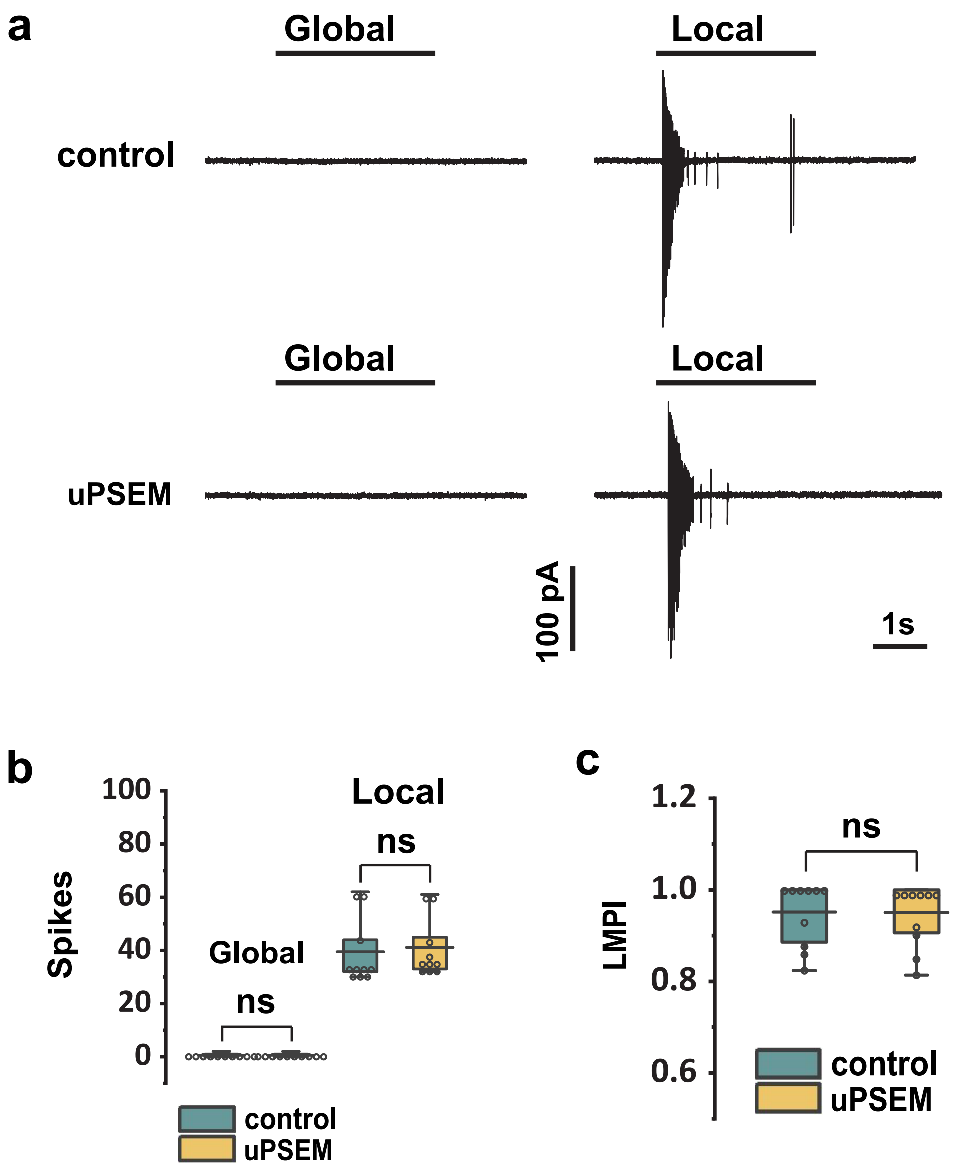

Supplementary Figure 7. Inactivation of COMS-AC has no effect on the OMS of W3 (UHD)-RGC. a. W3 (UHD)-RGC spiking during global and local motion in control and uPSEM^{792}. **b.** Effects of uPSEM^{792} on spiking rate. Global: ns ($p = 1$), local: ns ($p = 0.25$). N = 10 cells, Wilcoxon Signed Rank test, two tailed. **c.** Effects of uPSEM^{792} on LMPI. N = 10 cells, ns ($p = 1$), Wilcoxon Signed Rank test, two tailed. The box plots display the mean, 25th, and 75th percentiles, while the whiskers indicate the 1.5 interquartile range. Source data of **b** and **c** are provided as a Source Data file

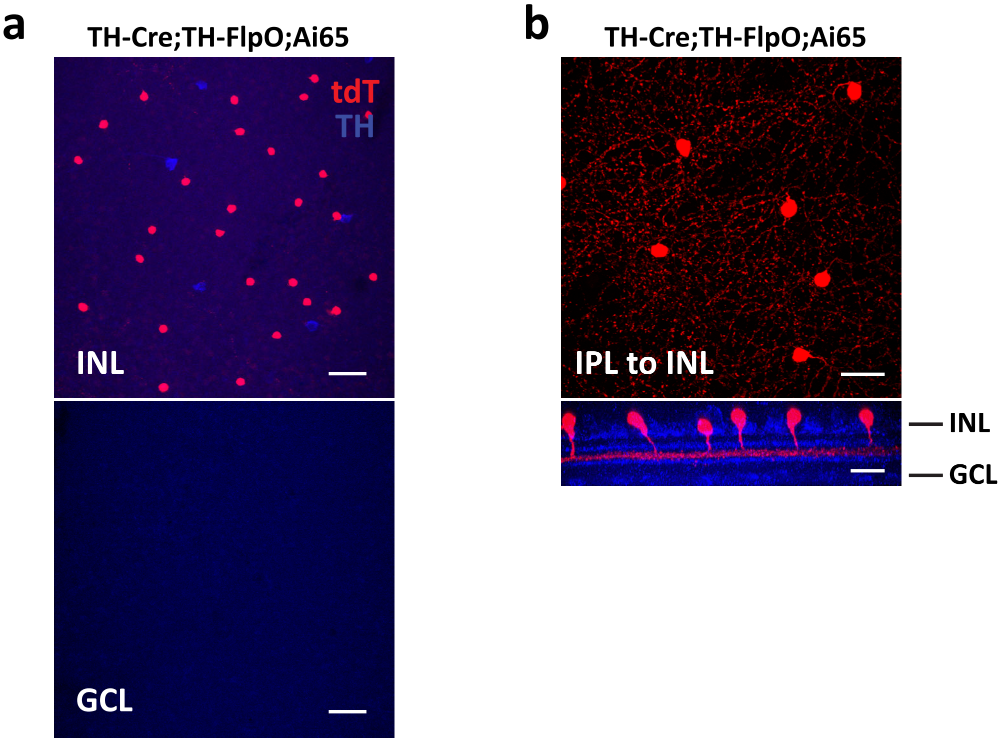

Supplementary Figure 8. Selective labeling of TH2-AC in TH-Cre;TH-2A-FlpO;Ai65 mice. a. Distribution of tdTomato-labeled cells in the INL and GCL. The flat mount retina was stained for tdTomato (red) and TH (blue). Scale bar: 50µm. **b.** Top: A projection of tdTomato labeled cells from IPL to INL to show their somas and processes. Bottom: A side view of td-Tomato-labeled cells with ChAT (blue) to show their uniform dendritic stratification. Scale bar: 20µm. Experiments were replicated independently in at least 7 retinae with similar results.

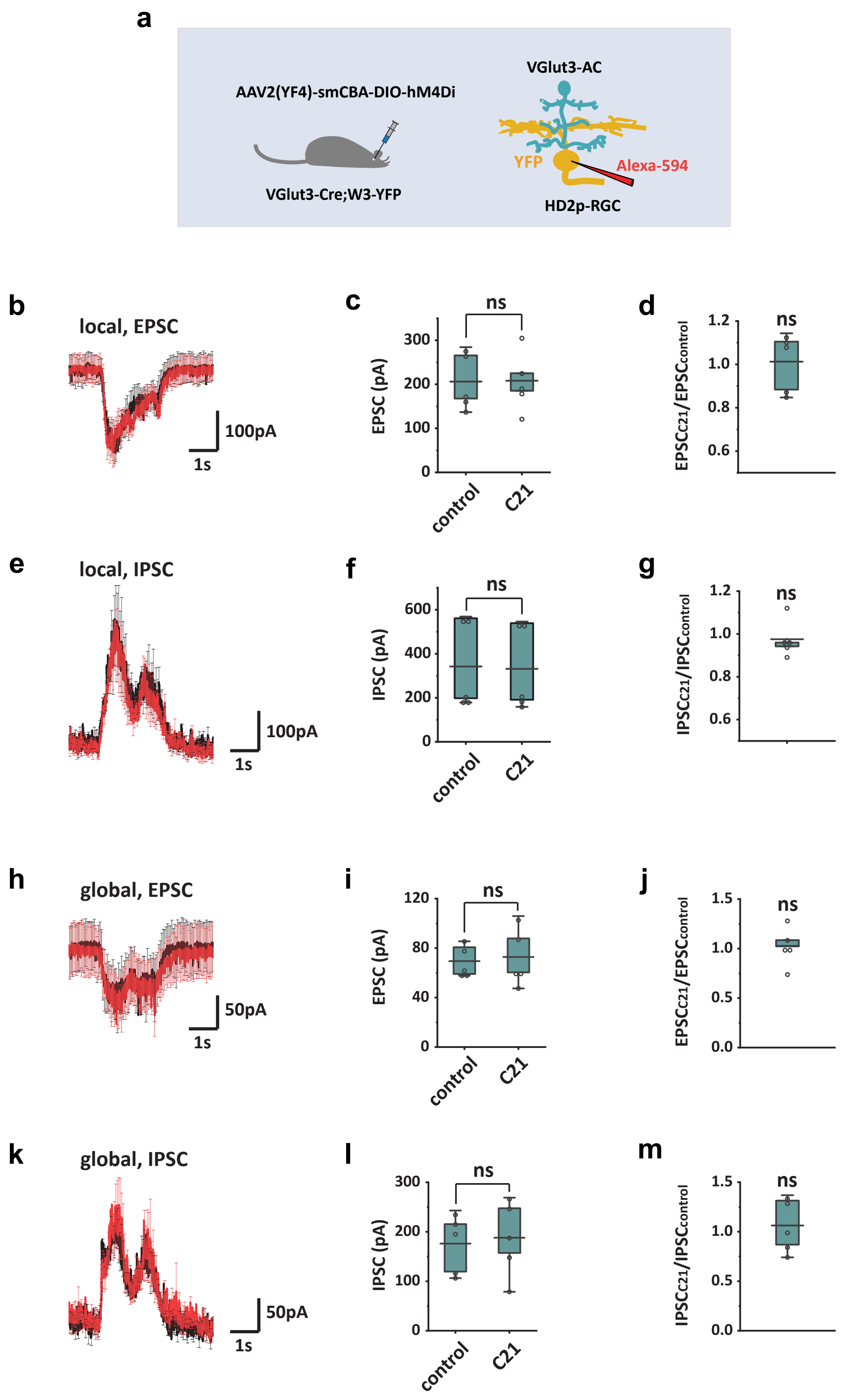

Supplementary Figure 9. Inactivation of VGlut3-AC has no effect on the OMS of HD2p-RGC. a. Schematic illustration for labeling VGlut3-AC with hM4Di and HD2p-RGC with YFP with intravitreal injection of AAV2(YF4)-CBA-DIO-hM4Di in VGlut3-Cre;W3-YFP mice. **b-m,** Effects of C21 on EPSC **(b-d)** and IPSC **(e-g)** of local motion, and EPSC **(h-j)** and IPSC **(k-m)** of global motion, n = 5 cells. p = 1 in **c,** p = 0.63 in **f,** p = 0.44 in **i,** p = 0.63 in **l,** Wilcoxon Signed Rank test, two tailed. p = 0.85 in **d,** p = 0.57 in **g,** p = 0.71 in **j,** p = 0.64 in **m,** one sample t test for mean = 1. The box plots display the mean, 25th, and 75th percentiles, while the whiskers indicate the 1.5 interquartile range. Source data of **c, d, f, g, i, j, l,** and **m** are provided as a Source Data file.
